# Transcranial Alternating Current Stimulation with Sawtooth Waves: Simultaneous Stimulation and EEG Recording

**DOI:** 10.3389/fnhum.2016.00135

**Published:** 2016-03-29

**Authors:** James Dowsett, Christoph S. Herrmann

**Affiliations:** ^1^German Center for Vertigo and Balance Disorders, Klinikum Grosshadern, Ludwig-Maximilians-UniversitätMunich, Germany; ^2^Experimental Psychology Lab, Center for Excellence “Hearing4all”, European Medical School, University of OldenburgOldenburg, Germany; ^3^Research Center Neurosensory Science, University of OldenburgOldenburg, Germany

**Keywords:** tACS, EEG, sawtooth, alpha, oscillations

## Abstract

Transcranial alternating current stimulation (tACS) has until now mostly been administered as an alternating sinusoidal wave. Despite modern tACS stimulators being able to deliver alternating current with any arbitrary shape there has been no systematic exploration into the relative benefits of different waveforms. As tACS is a relatively new technique there is a huge parameter space of unexplored possibilities which may prove superior or complimentary to the traditional sinusoidal waveform. Here, we begin to address this with an investigation into the effects of sawtooth wave tACS on individual alpha power. Evidence from animal models suggests that the gradient and direction of an electric current should be important factors for the subsequent neural firing rate; we compared positive and negative ramp sawtooth waves to test this. An additional advantage of sawtooth waves is that the resulting artifact in the electroencephalogram (EEG) recording is significantly simpler to remove than a sine wave; accordingly we were able to observe alpha oscillations both during and after stimulation. We found that positive ramp sawtooth, but not negative ramp sawtooth, significantly enhanced alpha power during stimulation relative to sham (*p* < 0.01). In addition we tested for an after-effect of both sawtooth and sinusoidal stimulation on alpha power but in this case did not find any significant effect. This preliminary study paves the way for further investigations into the effect of the gradient and direction of the current in tACS which could significantly improve the usefulness of this technique.

## Introduction

Transcranial alternating current stimulation (tACS) is increasingly being used as both an investigational tool and for clinical intervention as it can modulate cortical activity in a frequency specific manner and is thought to function by entraining neural oscillations. A number of studies have shown that tACS at alpha frequencies can enhance alpha oscillations (Zaehle et al., 2010; Neuling et al., 2013; Helfrich et al., 2014). The current study continues this line of research by observing the effect of 10 Hz tACS on alpha power.

A provisional explanation for the frequency specific effects of tACS is that ongoing neural oscillations are entrained to the electrical stimulation. One mechanism by which this might happen is that the applied electrical field modulates the local field potential such that the positive (anodal) phase of the stimulation increases the likelihood of neuronal spiking and the negative (cathodal) phase decreases the likelihood. As a result the ongoing neural oscillations may become synchronized with the alternating current; this has been shown to be the case in both recordings from cortical slices stimulated with an electrical field (Fröhlich and McCormick, 2010), and in intracranial recordings in rats stimulated with electrodes on the surface of the skull (Ozen et al., 2010).

The majority of tACS studies to date have used a sinusoidal waveform, however an alternating current does not have to be sinusoidal; it can be a square wave, triangular, pulsed or any arbitrary waveform. There have been a few exceptions to the convention of using sinusoidal waves for alternating or oscillating transcranial stimulation which have shown interesting results, for example pulsed current stimulation has been shown to affect corticospinal excitability (Jaberzadeh et al., 2014) and slow wave rectangular stimulation has been shown to have an effect on memory consolidation during sleep (Marshall et al., 2006).

There are various reasons why steep or instantaneous changes in current, such as in square waves or sawtooth waves, might be better suited to entraining ongoing neural oscillations. Fröhlich and McCormick (2010, Supplementary Material) have shown that ramps of increasing voltage with a steeper gradient resulted in increased neural firing *in vitro*, relative to ramps with a low gradient but which reached the same maximum voltage. This demonstrates that it is not only the total amount of current but also the rate of change of current which modulates neural firing.

To understand how electrical fields might entrain neural oscillations it is important to consider the mechanism behind different cortical rhythms. Reato et al. (2013) discuss how slow wave neural oscillations consist of a period of high activity followed by an inactive period; the duration of the high activity state is thought to be determined by the depletion of cellular resources and cannot be easily changed, whereas the duration of the low activity state can be more readily modulated and under certain conditions can be ended by a single spike at the optimal time resulting in a cascade of firing which begins at the next cycle of the oscillation. A relatively weak external electric current, with the optimal polarity and at the critical point in time, would be sufficient to initiate the onset of the active state, and when repeated at the right frequency might drive or entrain ongoing activity. We can speculate that a sudden change in current would be more suited to this role than the relatively slow rise of a sine wave if the transition from one state to another depends on a sufficient number of neurons firing together at a critical time.

By administering transcranial electrical stimulation with waveforms such as square wave or sawtooth waves the maximum rate of change of current flow at the cortex becomes more similar to other brain stimulation techniques such as transcranial magnetic stimulation (TMS) where the current flow in the cortex steeply rises and falls in less than a millisecond, although the mechanism of action is completely different. TMS is super-threshold, directly inducing action potentials whereas tACS is subthreshold, influencing the probability of action potentials. In addition, electroconvulsive therapy (ECT) is known to be significantly more efficient at inducing seizures with lower electrical charge using square waves rather than sine waves (Abrams, 2002); again the mechanism of action is entirely different, but if a sudden change in current is more effective than a sinusoidal current at causing neurons to fire it is not unreasonable to assume that the same is true for the subthreshold effect of much weaker currents on the probability of neurons firing.

**Figure 1:**
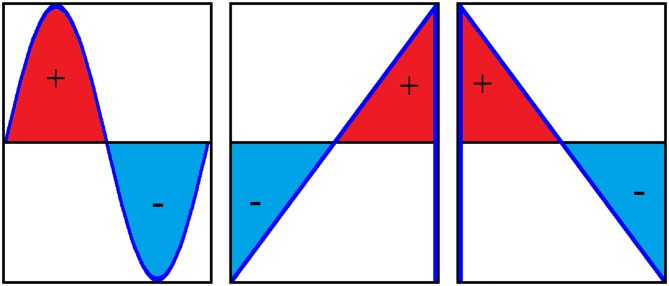
**One cycle of a sine wave, positive ramp sawtooth and negative ramp sawtooth (from left to right).** Positive and negative ramp sawtooth waves contain identical amounts of positive and negative charge, i.e., the area under the curve is the same.

**Figure 2:**
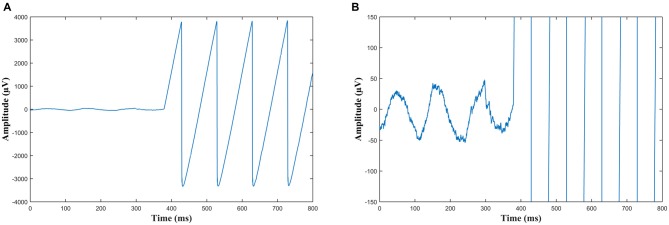
**(A)** An example of the onset of sawtooth wave Transcranial alternating current stimulation (tACS) recorded in EEG from electrode Pz. Note the sawtooth waves are slightly rounded at the peaks due to capacitance. **(B)** The same data as **(A)** but with the scale adjusted such that the ongoing alpha oscillations can be seen before the stimulation starts and are obscured during stimulation, which at this scale appears as near vertical lines.

In the current study, we chose to compare tACS with positive ramp and negative ramp sawtooth waves (Figure 1, example EEG data in Figure 2). A sawtooth wave consists of two distinct components: the linear ramp during which the current gradually changes over 100 ms (with 10 Hz stimulation), and the vertical transition where the current switches direction instantaneously. We chose to use sawtooth waves to differentiate between the effect of a sudden jump in polarity at the Oz electrode from anode to cathode in the case of positive ramp, and from cathode to anode in the case of negative ramp. A square wave would contain sudden transitions in both directions.

It is known from TMS studies that changing the current direction (by rotating the orientation of the coil) can have significantly different effect on the neural response. This variation has been shown in the motor cortex to be generally consistent across the majority of individuals, while a minority show a different optimal direction (Balslev et al., 2007). This is thought to be due to different populations of neurons being activated preferentially by different current directions. Variation in current direction has also been shown to affect TMS phosphene threshold; lateral to medial induced current in the visual cortex is optimal to induce phosphenes (Kammer et al., 2001). Interestingly, a recent study has shown that TMS evoked alpha oscillations, generated with the TMS coil held vertically such that the significant induced current in the cortex flows in the anterior–posterior direction, show the same pattern of variation in amplitude due to attentional shifts as spontaneous alpha oscillations (Herring et al., 2015); this current direction is comparable to the tACS in the current study, i.e., flowing between Oz and Cz.

If current direction and gradient are important, we hypothesized that positive and negative ramp sawtooth would have a different effect on alpha power. Conversely, if there were no difference in cortical activity this would suggest that the gradient of the current is irrelevant and any effect is simply due to the alternating periods of positive and negative current.

A further advantage of sawtooth waves is that the resulting artifact in the EEG recordings during stimulation is simpler to remove; the distinct properties of sawtooth waves, i.e., consisting of straight lines with a steep transition, do not occur in nature and as such are easily distinguishable from neural activity, especially in the frequency domain where they show characteristic harmonics. As such it is possible to be sure that no residual artifact remains in the cleaned data. It should be noted that this is also true for square waves which could also be analyzed in this way in future studies.

## Materials and Methods

### Experimental Procedure

Thirty healthy subjects (16 female) with a mean age of 25 (max: 30 min: 19) participated in the study. Participants gave written consent after being fully informed as to the experimental procedure. All participants self-reported as being right handed and free from neurological or psychiatric diseases. The experimental protocol was approved by the local ethics committee.

Each participant came into the lab on four separate days and received a different condition on each day: sinusoidal tACS, positive ramp sawtooth tACS, negative ramp sawtooth tACS and sham stimulation. The order of conditions was randomized. EEG was recorded for 5 min before stimulation, during the 10 min stimulation and for 5 min post stimulation.

All tACS had peak-to-peak amplitude of 2 mA and was administered from a stimulator with the option of delivering current controlled by a remote input (Eldith, Neuroconn, Ilmenau, Germany), the waveforms were generated in MATLAB (The MathWorks Inc., Natick, MA, USA) at 5000 Hz and sent to the stimulator via a digital-to-analog converter (National Instruments USB-6229 BNC). The stimulating electrodes were a 4 cm × 4 cm electrode centered on Oz and a 5 cm × 7 cm electrode centered on Cz. These sizes were chosen to give a higher current intensity over the occipital cortex, as this is thought to be a source of alpha oscillations, and a lower current intensity over Cz which is not thought to be involved in the generation of alpha oscillations. The polarity of the stimulation was such that when the input waveform was positive the electrode at Oz was anodal and Cz was cathodal, and* vice versa* in the negative half of the wave. All tACS was delivered at 10 Hz.

In the sham condition stimulation was delivered at full power (sine wave) for 10 s and then faded to zero over a further 10 s. Pilot data was collected from three lab members who reported that they could feel the sensation of tACS at onset but could no longer feel the on-going stimulation after 1 min, and could not distinguish between this and the sham condition, this suggested that this procedure is sufficient to induce the sensation of stimulation which persists for longer i.e., participants cannot tell when the stimulation ends.

On each experimental session the tACS electrodes were attached using a conductive paste and the impedance was measured to insure it was below 10 k ohms (in most cases it was below 5 k ohms). Next, the EEG cap was fitted over the tACS electrodes and five recording electrodes were set to the parietal sites (P7, P3, P_Z_, P4 and P8) according to the 10–20 System. The EEG was amplified using a BrainAmp amplifier (Brain Products, Munich, Germany). Impedance of the EEG electrodes was kept below 10 k ohms and was recorded with a sampling rate of 5000 Hz (the same as the tACS signal). The reference electrode was attached to the tip of the nose and a further electrode was placed below the right eye to record eye movements. The ground electrode was positioned on the forehead at electrode position Fpz. The experiment was performed in an electrically shielded, sound-proof, and dimly lit room (Vacuumschmelze, Hanau, Germany).

The experiment was double blinded in as much as the experimenter who attached the tACS electrodes, fitted the EEG cap and explained the procedure to the participant was not aware of the stimulation the participant would receive; the order of conditions was determined at random by the computer controlling the experiment and only observed by a second experimenter.

Throughout the entire experiment (pre, stimulation/sham and post EEG) the participants were instructed to fixate on an LED and press a response button whenever it illuminated to insure a consistent level of vigilance. The LED illuminated at random intervals between 50 and 60 s. We chose to record with eyes open and not with eyes closed because a previous study (Neuling et al., 2013) has shown an increase in alpha power after tACS with eyes open but not with eyes closed, so it would seem that tACS does not have a significant effect on eyes-closed alpha power, perhaps because of a ceiling effect.

After each experimental session participants were given a questionnaire to asses any possible adverse effects (Neuling et al., 2013) which asked about any of the following symptoms: headache, neck pain, scalp pain, tingling, itching, burning sensation, skin redness, sleepiness, trouble concentrating and acute mood change. Participants were asked to indicate the intensity of the side effect (1, absent; 2, mild; 3, moderate; 4, severe) and if they attributed this to the tACS. Additionally they were asked on each day if they felt the simulation and if so for how long they thought the stimulation lasted. Participants were also asked if they perceived phosphenes. The results of these questionnaires were collected and analyzed.

As an additional analysis, to test the artifact removal method, a 10 Hz sawtooth wave of comparable size to a typical artifact was generated in MATLAB and added to the 10 min EEG recording from the sham condition (excluding the 20 s stimulation) for each participant, the artifact was then removed using the same procedure (described below) and compared to the raw data.

### Data Analysis

Electrode Pz was initially selected for amplitude analysis as in previous studies (Neuling et al., 2013). For some of the participants Pz could not be used for the online analysis as the tACS artifact was too large and caused the signal to clip, rendering the data unusable, as such electrode P4 was used, as this was the only electrode not corrupted in all participants and all conditions. The same electrode was used across all conditions. While there is the possibility that any effect found might only be in the right hemisphere it is unlikely as the stimulating electrodes were positioned on the mid-line and we would expect the current to reach both hemispheres equally.

Of the 30 participants tested, 12 had no observable peak in the alpha band above 1/f noise in either the pre or post measurement. If there is no observable alpha activity we would not be able to see any change in alpha power and as such these participants were not included in any further analysis. Experience from other studies has shown that it is not uncommon for such a high percentage of participants to have no observable eyes-open alpha peak, for example Min et al. (2007) found that 8 out of 23 subjects had no detectable alpha peak, this is a similar ratio as found in the current study (12 out of 30). Other studies (e.g., Smit et al., 2006) have found a significant number of individuals showing no alpha peak, although a lower percentage than found in the current study. It is unfortunate that such a high number had no detectable alpha peak, however this data is still useful as we were able to remove the artifact and show that there is no residual peak at 10 Hz, demonstrating that the artifact removal method does not leave a residual artifact (see “Discussion” Section).

Of the 18 remaining participants three were rejected from further analysis due to excessive noise caused by the tACS or excessive muscular artifacts making further analysis impossible; as a result 15 participants (six female) were included in all subsequent analyses.

Artifact removal and pre-processing was performed with MATLAB and statistical tests were performed with R (R Foundation for Statistical Computing, Vienna, Austria).

### Artifact Removal

**Figure 3:**
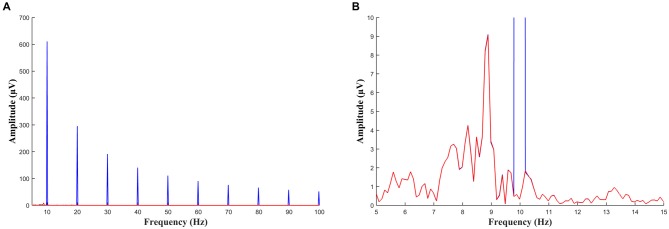
**(A)** FFT of a 10 s segment of EEG during sawtooth tACS before (blue) and after (red) the artifact has been removed. Large peaks at 10 Hz and at all harmonics of 10 Hz can be seen before the data is cleaned. **(B)** The same data as **(A)** with the scale adjusted such that the alpha peak can be seen. This participant had an individual alpha frequency of approximately 9 Hz. The data before and after artifact removal are virtually identical except for the peak at 10 Hz which has been removed in the cleaned signal (red).

The tACS artifact was removed using a modified version of a template subtraction technique used by Helfrich et al. (2014), a critical difference in the current study is that a template of 10 s was used to give a higher frequency resolution. The EEG recording during stimulation was first divided into 10 s segments. For each segment a 10 s sliding window was used, starting from 10 s before the period to be analyzed and moving forward in 100 ms steps (the length of one 10 Hz tACS oscillation) for 200 steps; these windows were then averaged to create a template of the artifact and subtracted from the original 10 s window (for example data, see Figure 3B).

The tACS stimulator delivers a constant peak current and as a result any changes in impedance will result in changes in the size of the artifact in the EEG recording (measured in micro-volts). Generally there is a gradual change in the size of the artifact over time due to the electrode gel drying out, participant sweating etc. In addition there are occasionally sudden jumps in the size of the artifact, most likely due to participant movement. For this method to work it is important that the tACS artifact does not change size suddenly during the period used to create the sliding window as this will result in an incorrectly sized template and a residual artifact in the cleaned data.

**Figure 4:**
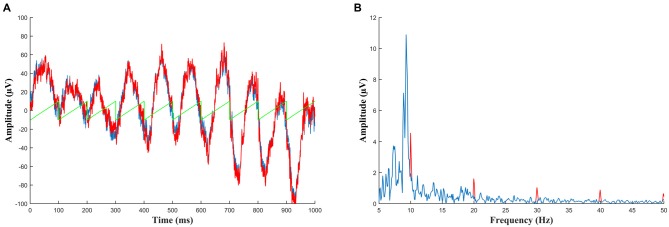
**(A)** Simulated data to demonstrate detection of a residual artifact. Ten seconds of baseline EEG (in blue, only 1 s shown) was added to a small sawtooth wave with amplitude of 10 μV (green) to create a corrupted signal (red) such as is seen when an incorrectly sized template is subtracted during artifact removal. Viewed in the time domain this signal cannot be differentiated from normal EEG. **(B)** The same data as **(A)** but viewed in the frequency domain. Here the corrupted signal (red) can easily be identified by harmonics which stand out above the level of noise. The peak at 10 Hz could potentially be entrained alpha oscillations but the other harmonics above 30 Hz (which continue throughout the frequency plot) indicate the presence of a sawtooth artifact. Therefore any segment which shows this activity should be rejected from analysis.

A number of steps were taken to insure against this by rejecting any segments for which the artifact was not correctly removed. Firstly, the template was created by averaging only sliding windows for which the amplitude at each data point was less than 200 μV above or below the amplitude of the segment to be cleaned. Secondly, before the template was subtracted, an FFT was performed on the template itself and it was not used if it contained activity at any frequency other than 10 Hz and harmonics (20 Hz, 30 Hz … etc.); this ensured that only consistent activity at exactly the stimulation frequency (±0.05Hz) would be subtracted (as a 10 s segment was used the resulting FFT had a resolution of 0.1 Hz). As a third step, the cleaned 10 s segment was rejected from any further analysis if it contained any evidence of residual artifact. A distinctive characteristic of sawtooth waves is that they contain strong harmonics when viewed in the frequency domain; with 10 Hz stimulation a sawtooth wave would show strong peaks at every multiple of 10 Hz (Figure 3A). Any 10 s segment which contained peaks at any multiple of 10 Hz above 20 Hz, greater than one standard deviation above the average level of noise in the adjoining ±5 Hz range, was rejected from further analysis (demonstrated with simulated data in Figures 4A,B). Although this is probably an overly conservative criterion (i.e., occasionally segments with no residual artifact but high levels of noise would have been rejected) it was selected to be certain that no residual artifact remained. The 20 Hz harmonic was not included in the rejection criteria as some participants showed beta peaks around 20 Hz in the pre-measurement and as such 20 Hz peaks could conceivably be entrained beta activity. Using a 10 s segment is preferable for this step as the harmonics which result from residual sawtooth artifact are more clearly visible above noise.

It should be noted that this method would not work with the sinusoidal stimulation as any residual artifact would only contain activity at 10 Hz with no harmonics and as such is not distinguishable from EEG at 10 Hz using only one electrode. For this reason, and because there were insufficient EEG electrodes for other artifact removal techniques such as PCA, the online data for the sinusoidal tACS was not analyzed as there would be no criteria for determining if the artifact had been fully removed.

As a final step the cleaned 10 s segments were further divided into 1 s segments and any containing eye blinks or muscular artifacts were rejected.

### EEG Analysis

The analysis of the cleaned online data, the offline data (the pre and post measurements) and the cleaned “simulated artifact” data was carried out using a modification of a method used by Zaehle et al. (2010). EEG data was split into 1 s segments, if a segment included an eye blink or muscular artifact it was rejected from further analysis.

The first 200 artifact free 1 s segments for pre, online and post for each condition were baseline corrected by subtracting the mean, multiplied by a hanning window, and an FFT was applied to each. The resulting FFT spectra were then averaged.

For each averaged spectra the peak value was taken as the maximum between 8 and 14 Hz. The amplitude of the alpha was taken as the mean of the range ±2 Hz from this peak. To account for individual/inter-trial variation in alpha amplitude each online and post alpha amplitude value was normalized relative to the average amplitude from the corresponding 5 min pre measurement. These relative values were then subjected to statistical analysis.

For the online data a repeated measures analysis of variance (ANOVA) with one factor of condition and three levels (positive ramp sawtooth tACS, negative ramp sawtooth tACS and sham) was performed on the normalized alpha amplitude values. For the post data a repeated measures ANOVA with one factor of condition and four levels (sinusoidal tACS, positive ramp sawtooth tACS, negative ramp sawtooth tACS and sham) was performed on the normalized alpha amplitude values. *Post hoc* pairwise *t*-tests with Bonferroni correction were carried out to compare conditions.

In addition, a repeated measures ANOVA with four levels was applied to the mean peak alpha amplitude of the 5 min pre-measurement for each condition to test for any significant differences between conditions (as each condition was recorded on separate days and alpha power can change from one day to the next).

## Results

### EEG Data

**Figure 5:**
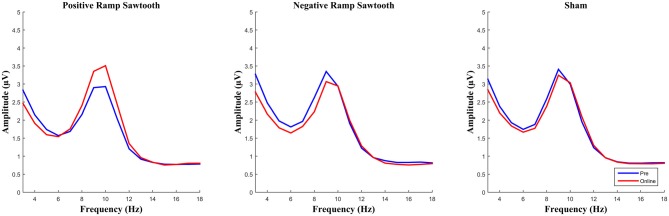
**Average spectra for all participants for pre measurement (blue) and online (red), before normalization.** Only the positive ramp sawtooth yielded a significant difference from sham after normalization.

EEG spectra comparing the amplitude of the alpha frequency band prior to stimulation to online data during stimulation (before normalization) are shown in Figure 5. For the online data a repeated measures ANOVA of the normalized alpha amplitudes revealed a significant effect of condition (*F*_(2,28)_ = 8.4735, *p* = 0.0013). Pairwise *t*-tests (Bonferroni corrected) showed a significant difference between positive ramp sawtooth and sham (*p* = 0.0059, cf. Figure 7), but no significant differences between any other conditions (*p* > 0.1).

**Figure 6:**
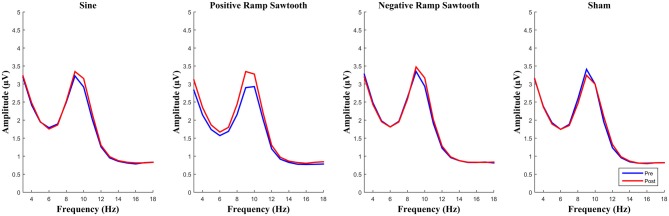
**Average spectra for all participants for pre (blue) and post (red) measurement, before normalization.** None of the differences between conditions reached significance after normalization.

EEG spectra before normalization comparing the amplitude of the alpha frequency band prior to stimulation to post stimulation are shown in Figure 6. For the post data a repeated measures ANOVA of the normalized alpha amplitudes showed no significant effect of condition (*F*_(3,42)_ = 2.01, *p* = 0.126). Pairwise *t*-tests (Bonferroni corrected) showed the difference between positive ramp sawtooth and sham to be approaching significance (*p* = 0.098), whereas *p* > 0.5 for all other condition pairs (cf. Figure 8).

**Figure 7:**
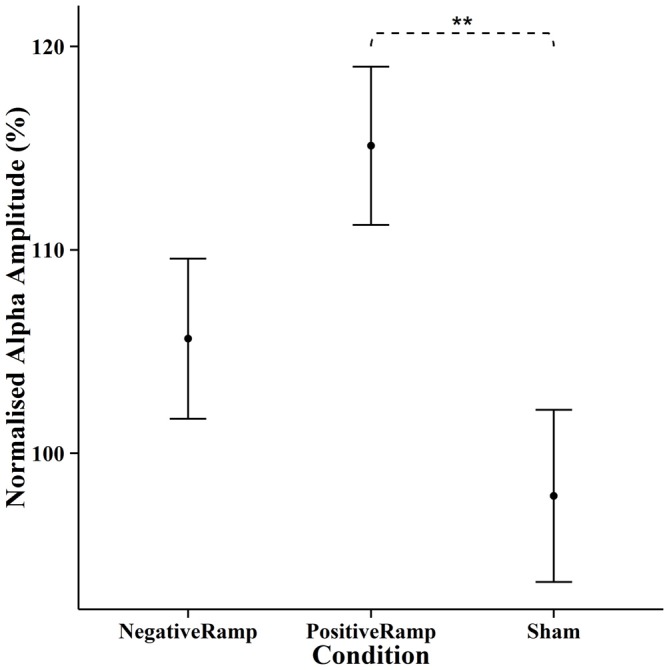
**Normalised mean alpha amplitude online for each condition, error bars show ±1 standard error of the mean.** Stimulation with positive ramp yielded a significantly stronger amplitude of alpha oscillations during stimulation compared to sham. “**” indicates *p* < 0.01.

**Figure 8:**
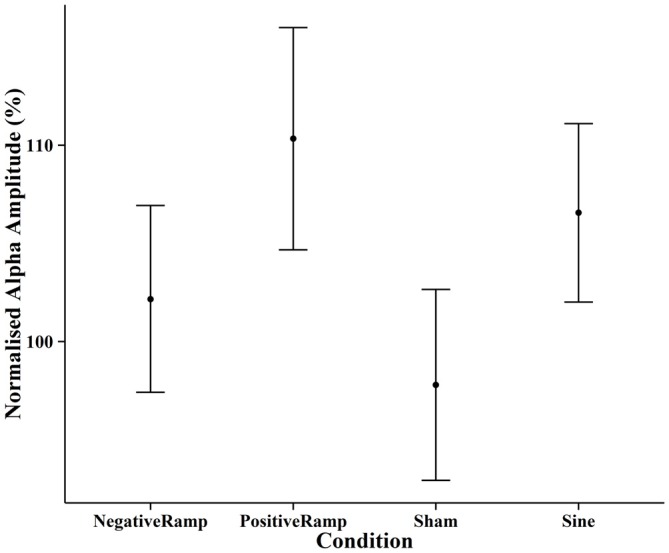
**Normalised mean alpha amplitude post-stimulation for each condition, error bars show ±1 standard error of the mean.** None of the differences between conditions reached significance for the pre-post comparison.

The repeated measures ANOVA comparing the mean alpha peak of the four pre-measurements showed no significant difference between the four conditions (*F*_(3,42)_ = 0.045, *p* = 0.987).

For the simulated artifact test the raw EEG from the sham condition was compared to the same data with a sawtooth artifact added and then removed; the resulting mean spectra were identical at all frequencies except 10 Hz where there were slight differences (<1%). The pairwise linear correlation coefficient between every cleaned 1 s segment of EEG data and the corresponding original data was calculated, the mean correlation was 0.97. The alpha peak of the mean FFT of the cleaned data was always either identical or slightly lower than the alpha peak of the original EEG, the mean error was 0.015 μV/Hz lower (the maximum error was 0.07 μV/Hz lower). Importantly, any error was always below the true value (because activity at 10 Hz is removed) and as such the increase in alpha amplitude found in the real data would at worst be an underestimate i.e., the true alpha power might be slightly higher.

### Questionnaire

All 30 participants were used for the analysis of the side-effects and sensation reports. Individual responses to each item on the questionnaire for each condition were entered into a Friedman test; there was no significant effect of condition for any of the side-effects (*p* > 0.1 for all). The most common reported sensations were Itching, Tingling and Heating (mean scores for all conditions <2, i.e., mild sensation). When asked to estimate how long the stimulation lasted 17 of the participants reported that they felt the stimulation for under a minute in all conditions, five reported the sensation of stimulation throughout the experiment in all conditions, four reported no sensation at all in any of the conditions and three were able to distinguish between the sham and stimulation conditions reporting sensation throughout the experiment in all conditions except sham. Therefore, all but three participants were successfully shamed in one way or another. Only these three participants reported seeing phosphenes throughout the experiment, there was no difference between the reports of phosphenes between any of the stimulation conditions. Importantly the side effect scores and estimates of stimulation duration were almost identical for positive and negative ramp sawtooth stimulation for all participants; as such the main finding of a difference between positive and negative ramp sawtooth waves (compared to sham) cannot be attributed to skin sensation or phosphenes (see, “Discussion” Section).

## Discussion

The primary aim of this study was to compare the effect of positive and negative ramp sawtooth wave tACS on alpha oscillations. The fact that it is possible to tell whether artifacts from non-sinusoidal tACS have been successfully removed is an additional advantage. Our main finding was that positive ramp sawtooth stimulation significantly increased alpha power during stimulation relative to a baseline condition, whereas negative ramp sawtooth did not. The positive and negative ramp sawtooth waves were identical in terms of frequency, peak current and total charge delivered (i.e., the derivative of current by time in coulombs); this indicates that the gradient of the current and the current direction play an important role in the modulation of ongoing alpha oscillations. As a sudden change in current is more likely to have an effect than a gradual change we can hypothesize that the steep change from 1 mA anodal to 1 mA cathodal at electrode Oz every 100 ms is likely to be the primary cause of the increase in alpha power found here. As both positive and negative ramp sawtooth waves contain a sudden change in current direction we can conclude that it is a sudden change in current in the optimal direction which is causing the effect.

**Figure 9:**
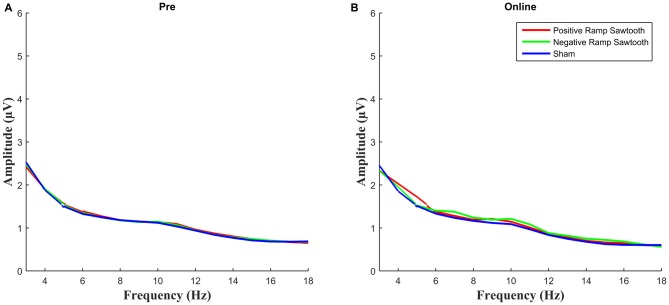
**Average spectra for all participants who showed no alpha peak in all conditions: (A) for the 5 min pre measurement, (B) for the 10 min of stimulation after the artifact has been removed**.

Participants with no observable peak in the alpha range did not show any peak during stimulation after the artifact was removed (Figure 9); this is further evidence that the artifact removal method does not leave any residual artifact.

Unfortunately we were not able to directly compare the online effect of sinusoidal tACS with the two sawtooth tACS conditions. Recent studies have had some success removing the artifact resulting from sinusoidal tACS in EEG (Helfrich et al., 2014), using a combination of template subtraction and PCA to remove any residual artifact, and in MEG (Neuling et al., 2015) using beamforming. However, it should be noted that careful observation of the size of the artifact in the EEG from the current study reveals that as well as the artifact changing size over time it can in some cases change in a different direction across electrodes (i.e., shrink in one electrode and grow in another) thus changing the topography of the artifact at the scalp; this would not be immediately obvious and as such caution should be used when interpreting data where a sinusoidal tACS artifact has been removed from EEG, even after PCA has been used to remove any residual artifact.

The ability to remove the tACS artifact from a single electrode (albeit by rejecting corrupted segments) is an advantage as it is simpler to setup and may be more desirable in some situations, for example in clinical settings where a full cap of 64 EEG electrodes is not practical.

In the test of the artifact removal method the simulated sawtooth artifact was removed almost perfectly from the 10 min EEG recording with only a slight loss at the stimulation frequency. This illustrates a drawback of the template subtraction method as used here: neural oscillations at exactly the stimulation frequency can also be included in the template and subtracted. As we used a 10 s template, only constant oscillations between 9.95 Hz and 10.05 Hz would be affected. There could potentially be neural oscillations entrained to exactly the stimulation frequency that would be lost. This can be demonstrated by adding a simulated artifact at 10 Hz to EEG data containing a steady state visually evoked potential (SSVEP), also at exactly 10 Hz, and removing the artifact; in this case the SSVEP would be lost (data not shown). However, as demonstrated in the simulated data, the frequency amplitude of the cleaned data (after artifact removal) is only ever slightly reduced at the frequency of stimulation, and never increased, and as such we can be confident that the increase in alpha amplitude found during the positive ramp sawtooth (relative to sham) is at worst a slight underestimate of the true alpha amplitude (if the true alpha amplitude were higher the effect would be more significant). Variations on the template subtraction method which overcome this limitation by creating the template from non-regular or pulsed oscillations (in a calibration phase prior to the regular tACS) are being investigated and will be discussed in future studies.

None of the stimulation conditions showed a significant effect on alpha power in the 5 min post-stimulation relative to the 5 min pre-stimulation. While other studies have found a significant after-effect of tACS on alpha power there are a number of differences in the experimental design which may explain why the current study did not show such an effect. Firstly we stimulated at 10 Hz rather than adjusting the frequency of the stimulation to the individual alpha frequency of the participant as other studies have done (Zaehle et al., 2010; Neuling et al., 2013). Secondly, we only stimulated for 10 min whereas other studies showing an after effect have applied stimulation for twenty minutes (Neuling et al., 2013; Helfrich et al., 2014). Zaehle et al. (2010) found an after-effect after 10 min of stimulation but with stimulation at individual alpha frequency and a different electrode montage to the one used here. Helfrich et al. (2014) used stimulation at 10 Hz but stimulated for twenty minutes. This would imply that the sustained increase in alpha power after stimulation is dependent on either the stimulation frequency matching the individual’s alpha frequency and/or stimulation with a duration of more than 10 min.

Blinding is an on-going problem for all transcranial electrical stimulation research. As stated in the results, 17 of the 30 participants reported that they felt the stimulation for under a minute in all conditions, indicating that the sham was successful. However, the problem remains that some individuals are more sensitive to the sensation of tACS and were not successfully shammed. Other studies (Zaehle et al., 2010; Neuling et al., 2013) have adjusted the current intensity to the threshold of skin sensation for each individual rather than using a fixed current intensity. Adjusting the current intensity to each individual’s threshold of skin sensation is problematic because of the large variation in sensitivity to tACS across participants, as demonstrated by the wide variety of reports of sensation in the current study; different current intensities should not be compared as they may be having different effects on the cortex. This is especially important when considering the results of Moliadze et al. (2012) who showed that tACS can inhibit cortical excitability at low intensity and switch to excitation when the intensity is increased. There is no reason why the sensitivity of the scalp would correlate with the effect of the tACS on the cortex; therefore it is better to keep the intensity constant and control for sensation in some other way such as a control site or different stimulation parameters. Importantly, we found no difference between the sensation of positive and negative ramp sawtooth waves, as these were the two conditions we were comparing. Our results show a significant difference between positive ramp sawtooth stimulation and no stimulation, and no significant difference between negative ramp sawtooth and no stimulation. So in this context the sham condition can be considered a baseline condition. This finding may prove useful for future research because the effect of positive and negative ramp sawtooth waves is different, but the sensation is identical, and could therefore serve as a better control condition in future studies as the frequency, current density and skin sensations are identical for the two waveforms (albeit still requiring a baseline condition).

## Conclusion

The ability to stimulate with waveforms other than sinusoidal is an important addition to modern tACS stimulators, both because sinusoidal waveforms may not be optimal for entraining neural oscillations and because more can be learnt about the underlying mechanisms of transcranial electrical stimulation by systematically varying parameters such as the gradient of the electrical current. This preliminary investigation demonstrates that enhancement of alpha oscillations can be observed during positive ramp sawtooth stimulation, that the sawtooth artifact can be removed from single electrodes, and that sawtooth waves are not significantly different to sinusoidal stimulation in terms of side effects. Additionally, our results imply that current direction and gradient are important factors to consider in the design of tACS protocols. Further studies are needed to tell if this effect is frequency specific as well as if other waveforms, such as square wave, could also be useful variants of tACS.

## Author Contributions

JD and CSH contributed to all aspects of the manuscript.

## Conflict of Interest Statement

The authors declare that the research was conducted in the absence of any commercial or financial relationships that could be construed as a potential conflict of interest.
